# 

**Published:** 1853-10

**Authors:** 


Vol. I. OCTOBER, 1853. No. 3.

### IOWA MEDICAL JOURNAL

CONDUCTED BY THE FACULTY OF THE

**Medical Department of the Iowa University.**

TERMS--TWO DOLLARS, IN ADVANCE.

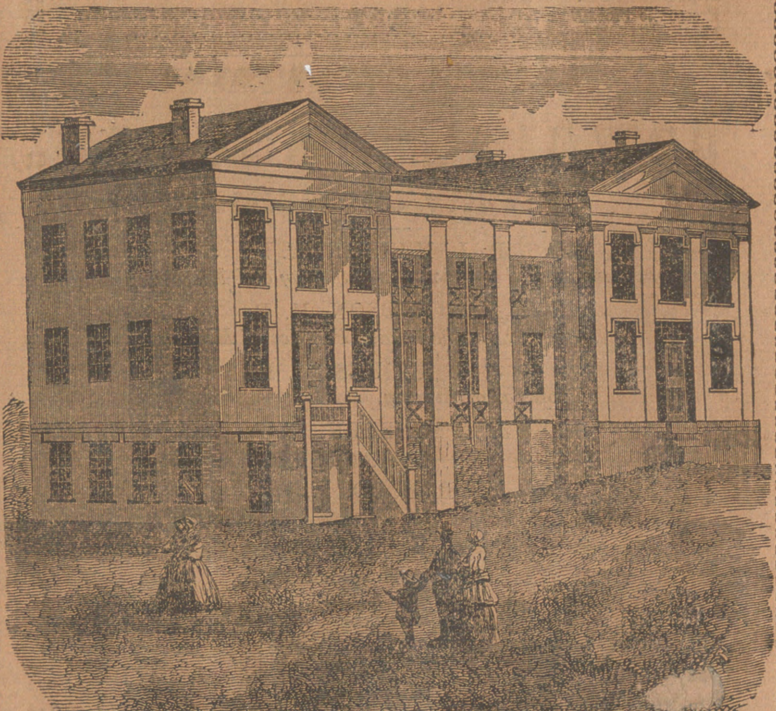

KEOKUK, IOWA:

Printed at the Whig Book and Job Office.

☞ ADDRESS ALL COMMUNICATIONS, POST-PAID, TO "MEDICAL COLLEGE, BOX 109, KEOKUK, IOWA." ☜

